# Hypervalent organoiodine compounds: from reagents to valuable building blocks in synthesis

**DOI:** 10.3762/bjoc.14.128

**Published:** 2018-06-21

**Authors:** Gwendal Grelier, Benjamin Darses, Philippe Dauban

**Affiliations:** 1 Institut de Chimie des Substances Naturelles, CNRS UPR 2301, Université Paris-Sud, Université Paris-Saclay, 1, av. de la Terrasse, 91198 Gif-sur-Yvette, France

**Keywords:** atom-economy, couplings, hypervalent iodine, oxidation, tandem reactions

## Abstract

Most of the polyvalent organoiodine compounds derive from iodoarenes, which are released in stoichiometric amounts in any reaction mediated by λ^3^- or λ^5^-iodanes. In parallel to the development of solid-supported reagents or reactions catalytic in iodine, a third strategy has emerged to address this issue in terms of sustainability. The atom-economy of transformations involving stoichiometric amounts of λ^3^- or λ^5^-iodanes, thus, has been improved by designing tandem reactions that allows for incorporating the aryl motif into the products through a subsequent one-pot nucleophilic addition or catalytic coupling reaction. This review summarizes the main achievements reported in this area.

## Introduction

Synthetic applications of the hypervalent iodine chemistry have grown exponentially in the last four decades as highlighted by several books and comprehensive reviews dedicated to this topic [1–13]. A still growing number of λ^3^- and λ^5^-iodanes are now available as non-toxic and environmentally benign reagents that allow for performing a wide range of transformations under mild conditions. The oxidation of functional groups, halogenations, C–C, C–O, or C–N couplings, dearomatization of phenols, rearrangements, to name but a few, have been reported using these compounds thereby reflecting their versatility.

**Scheme 1:**
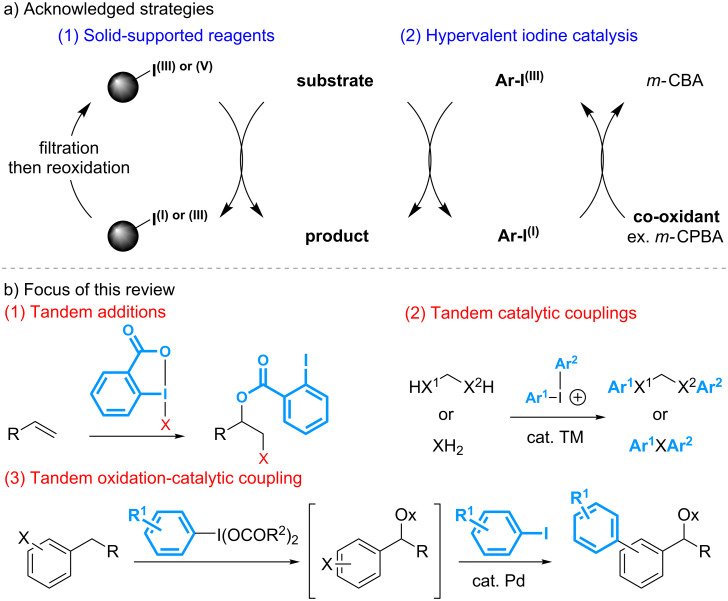
Strategies to address the issue of sustainability with polyvalent organoiodine reagents.

A survey of the general structure of polyvalent organoiodine compounds reveals that they are prepared mostly from iodoarene starting materials. Accordingly, any transformation relying on stoichiometric amounts of these reagents will inevitably produce the same quantity of iodoarene derivatives, which can be considered as a waste in the context of sustainable chemistry. Various strategies, thus, have been described to address this issue. A first solution has arisen from the development of polystyrene-supported reagents, which allows their recycling following simple filtration and re-oxidation (reaction 1 in Scheme 1a) [14–15]. More recently, the design of processes catalytic in iodine compounds has been extensively investigated and significant achievements have been reported making iodine compounds now useful organocatalysts in asymmetric synthesis (reaction 2 in Scheme 1a) [15–24].

In parallel to these investigations, a third strategy has been envisaged with the development of tandem reactions involving a step that enables the introduction of the iodoarene side-compound into the products. Various sequences combining oxidation reactions, nucleophilic additions, or aromatic couplings, thus, have been reported (Scheme 1b). In addition to address the issue of sustainability, this complementary solution has provided opportunities to explore a new chemical space. This review aims to highlight the main achievements reported in this context since the first study described in 1995 on the palladium-catalyzed cross couplings of symmetrical diphenyl-λ^3^-iodanes with sodium tetraphenylborate [25].

## Review

### Tandem additions

λ^5^-Iodanes such as the Dess–Martin periodinane or IBX [26], and λ^3^-iodanes such as benziodoxolones [27], are versatile reagents in organic synthesis. These are often used, respectively, for the oxidation of alcohols or carbonyl compounds, and in atom-transfer reactions. These transformations always lead to the release of 2-iodobenzoic acid in the reaction mixture. Thus, with the aim to value this side product, a variety of tandem reactions including an oxidation and an addition step have been designed to incorporate the nucleophilic acid into the final product.

#### λ^5^-Iodane reagents

**Scheme 2:**
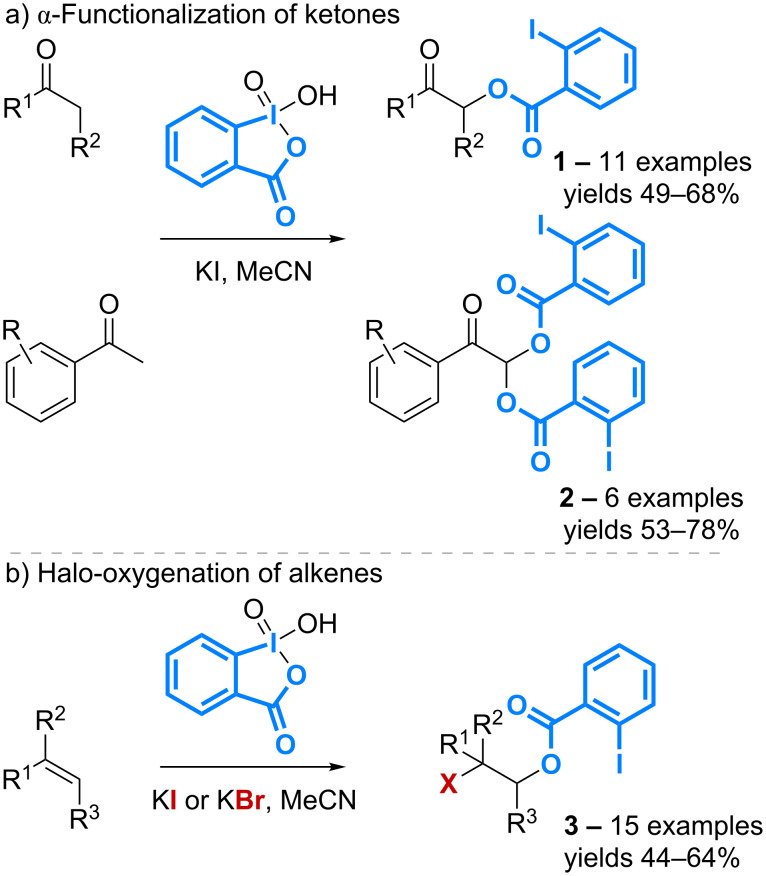
Functionalization of ketones and alkenes with IBX.

One of the first studies documenting the use of the iodoarene moiety as a building block in synthesis has been reported in 2004. The λ^5^-iodane reagent IBX has been shown to promote the α-functionalization of ketones following the introduction of the 2-iodobenzoic acid motif (Scheme 2a) [28].

Mono- and disubstituted products **1** and **2** are obtained respectively from aliphatic ketones and methyl aryl ketones. The reaction conditions have then been extended to the functionalization of alkenes to afford 1,2-halo-oxygenated compounds **3** (Scheme 2b) [29]. This transformation has recently been used for the stereoselective *trans* iodo-benzoylation of glycals using a combination of IBX and molecular iodine, that is considered as a source of I^+^ formed from the in situ generated hypoiodite species [30].

**Scheme 3:**
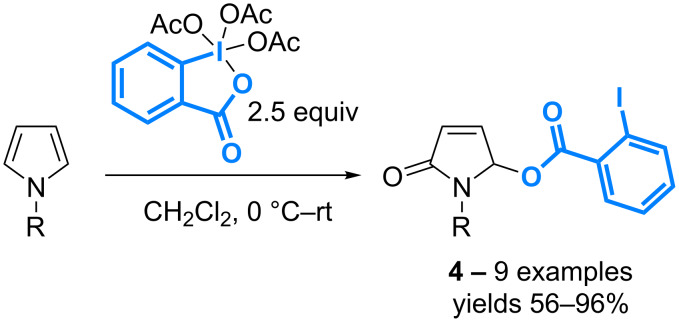
Functionalization of pyrroles with DMP.

The controlled oxidation of various *N*-(alkyl)- and *N*-(aryl)pyrroles with Dess–Martin periodinane also leads to introduction of the iodobenzoic acid motif onto the pyrrole ring. 5-Aroyloxy-γ-lactams **4** can be isolated with yields in the 56–96% range, however, the reaction requires 2.5 equivalents of the λ^5^-iodane reagent, a result that supports its limited efficiency in terms of atom economy (Scheme 3) [31].

#### λ^3^-Iodane reagents: CF_3_-benziodoxolone (Togni’s reagent)

The ability to introduce the 2-iodobenzoic acid motif released from benziodoxolones has first been noticed by Ochiai in an isolated example of radical benzoyloxylation of THF [32], and by Gouverneur in the study of trifluoromethylation of allylsilanes [33]. The use of this acid as an oxygen nucleophile, then, has been more fully investigated by the groups of Szabó and Sodeoka who have simultaneously described the copper-catalyzed benzoyloxy-trifluoromethylation of alkenes and alkynes using Togni’s reagent **5**.

**Scheme 4:**
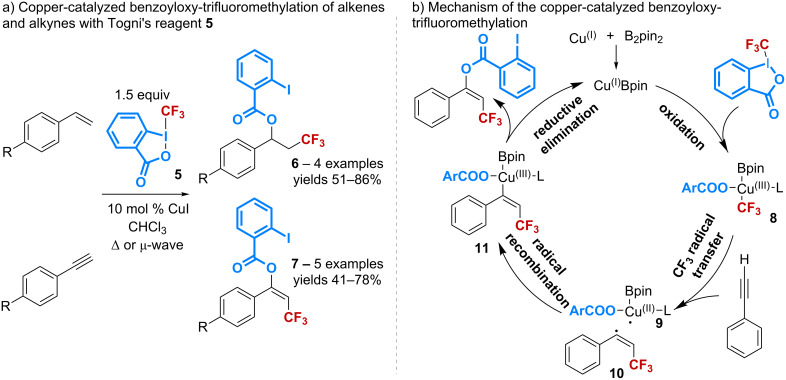
Catalytic benzoyloxy-trifluoromethylation reported by Szabó.

The group of Szabó has first reported the use of copper(I) iodide as a catalyst for the regioselective difunctionalization of aromatic alkynes and alkenes with an optimal atom-economy (Scheme 4a) [34–35]. The transformation proceeds efficiently, particularly in the presence of an electron-donating substituent on the aromatic ring. The same reaction has been later found to be mediated by copper(I) cyanide starting from *p*-methoxystyrene [36]. However, under these conditions, other styrene derivatives bearing a phenyl, a *tert*-butyl, or an electron-withdrawing substituent have been shown to afford products resulting from a cyanotrifluoromethylation reaction.

The mechanistic study of the oxy-trifluoromethylation of phenylacetylene has then led to demonstrate that the reaction is accelerated in the presence of additives such as B_2_pin_2_ [35]. A mechanism involving an initial step of transmetallation of B_2_pin_2_ with the Cu(I) catalyst was proposed (Scheme 4b). The intermediate Cu–Bpin, then, could undergo an oxidative addition into the CF_3_–I bond to give **8** that, after a CF_3_ radical transfer, would afford the radicals **9** and **10**. Radical recombination followed by reductive elimination would finally lead to the *E*-product and regenerate the Cu–Bpin complex.

**Scheme 5:**
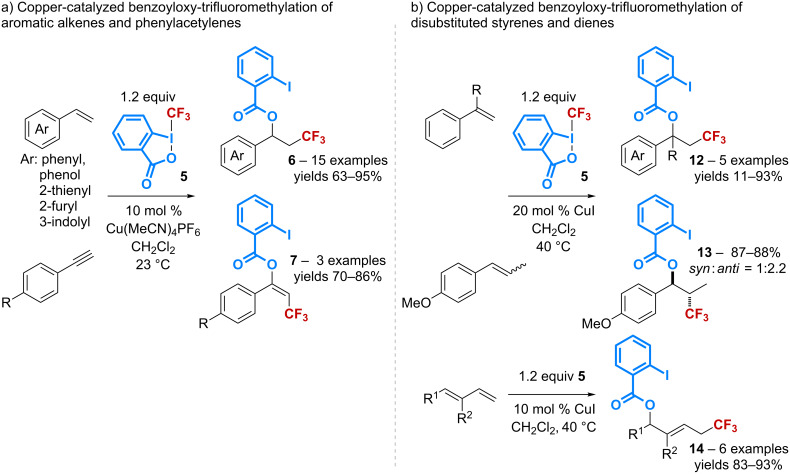
Catalytic benzoyloxy-trifluoromethylation reported by Mideoka.

The group of Sodeoka, in parallel, has described the same 1,2-difunctionalization reaction of alkenes and alkynes with **5** in the presence of [Cu(MeCN)_4_]PF_6_ as the catalyst (Scheme 5a) [37]. It should be pointed out that this copper(I) complex was previously described by Szabó as a poor catalyst in his process. The reaction applies to phenylacetylenes and various types of aromatic alkenes, however, its scope has been extended to dienes and disubstituted styrenes by using the less Lewis acidic CuI complex (Scheme 5b) [38]. Interestingly, *cis* and *trans* β-methyl-styrenes lead to product **14** with the same *syn*:*anti* ratio while 1,4-addition products containing an (*E*)-olefin are selectively obtained from dienes. The authors have also shown that the reaction performed in the presence of a stoichiometric amount of *p*-TsOH gives β-trifluoromethylstyrene derivatives instead of the expected oxy-trifluoromethyl compound.

**Scheme 6:**
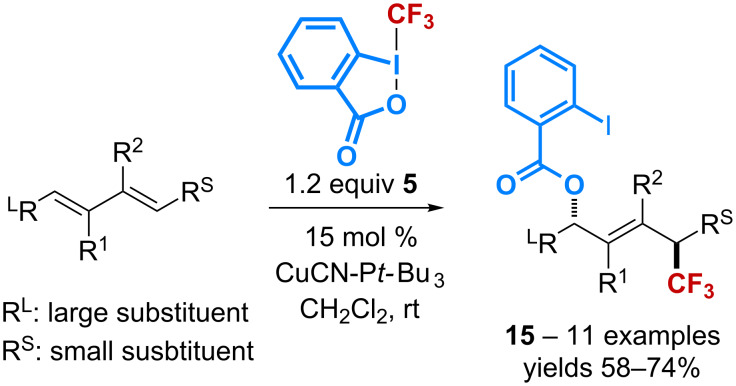
Catalytic 1,4-benzoyloxy-trifluoromethylation of dienes.

The benzoyloxy-trifluoromethylation of dienes has also been reported with CuCN as the catalyst. The reaction, again, is selective with respect to the olefin geometry – (*E*)-alkenes are exclusively obtained – and the regioselectivity – products resulting from a sterically-controlled 1,4-*anti*-addition are selectively isolated (Scheme 6) [39]. The proposed radical mechanism, which takes into account the beneficial effect of the bulky monophosphine P(*t-*Bu)_3_ on the reaction rate, is consistent with that reported by Szabó (Scheme 4b).

**Scheme 7:**
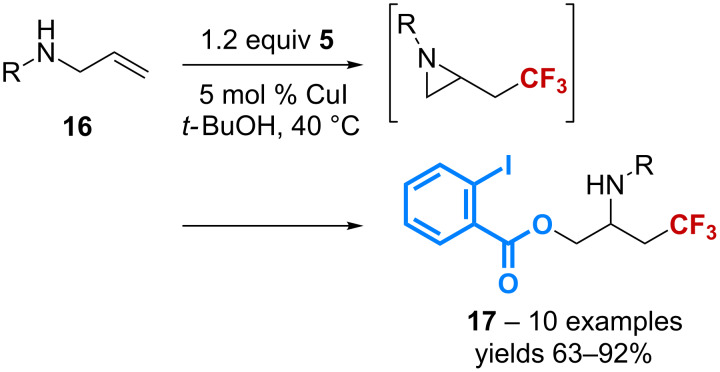
Catalytic benzoyloxy-trifluoromethylation of allylamines.

The scope of the copper-catalyzed benzoyloxy-trifluoromethylation has been extended to several other substrates. Starting from *N-*(aryl)- and *N-*(benzyl)allylamines **16**, the reaction affords β-benzoyloxy-β’-trifluoromethylamines **17** through the formation of an aziridine intermediate (Scheme 7) [40]. The latter was further utilized to give access to a variety of β-trifluoromethylamines after reaction with several *O-*, *N*-, *S*-, and *C*-nucleophiles.

**Scheme 8:**
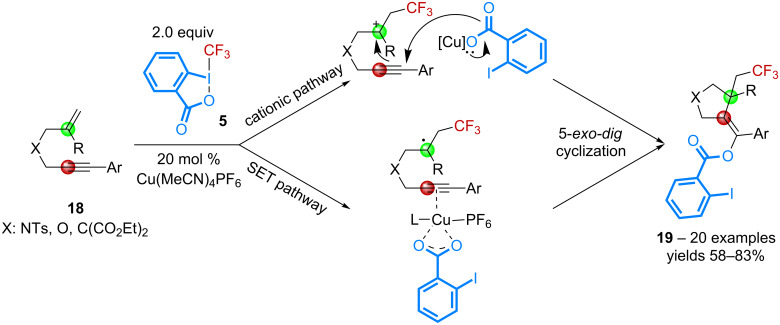
Catalytic benzoyloxy-trifluoromethylation of enynes.

Application of the same reaction conditions to enynes **18** has led to the discovery of an elegant cascade that gives access to a wide range of trifluoromethylated five-membered carbo- and heterocycles **19** (Scheme 8) [41]. Six-membered heterocycles can also be obtained in good yields. The reaction is believed to involve either a tertiary radical intermediate or a carbocationic species, both being able to undergo a 5-*exo-dig* type cyclization to afford compounds **19**.

**Scheme 9:**
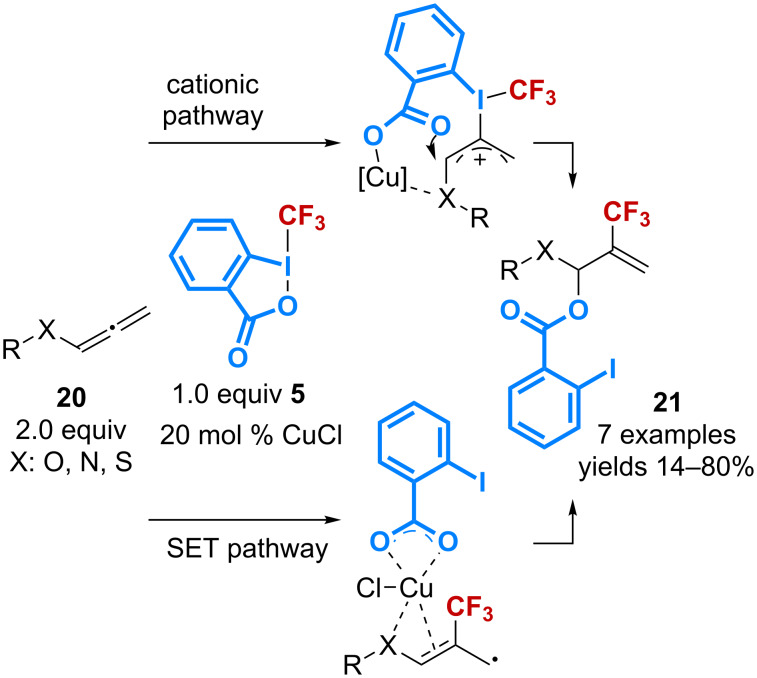
Catalytic benzoyloxy-trifluoromethylation of allenes.

The copper-catalyzed benzoyloxy-trifluoromethylation has also been applied to the conversion of allenes **20**. A regioselective 1,2-addition on the internal π-bond was observed to afford the products **21**, because of the presence of a heteroatom substituent that can stabilize the radical or cationic intermediate by coordination with the copper complex (Scheme 9) [42]. However, the efficiency of the reaction is limited by the need to use 2 equivalents of allenes **20** to obtain good yields.

#### λ^3^-Iodane reagents: alkynylbenziodoxolone

**Scheme 10:**
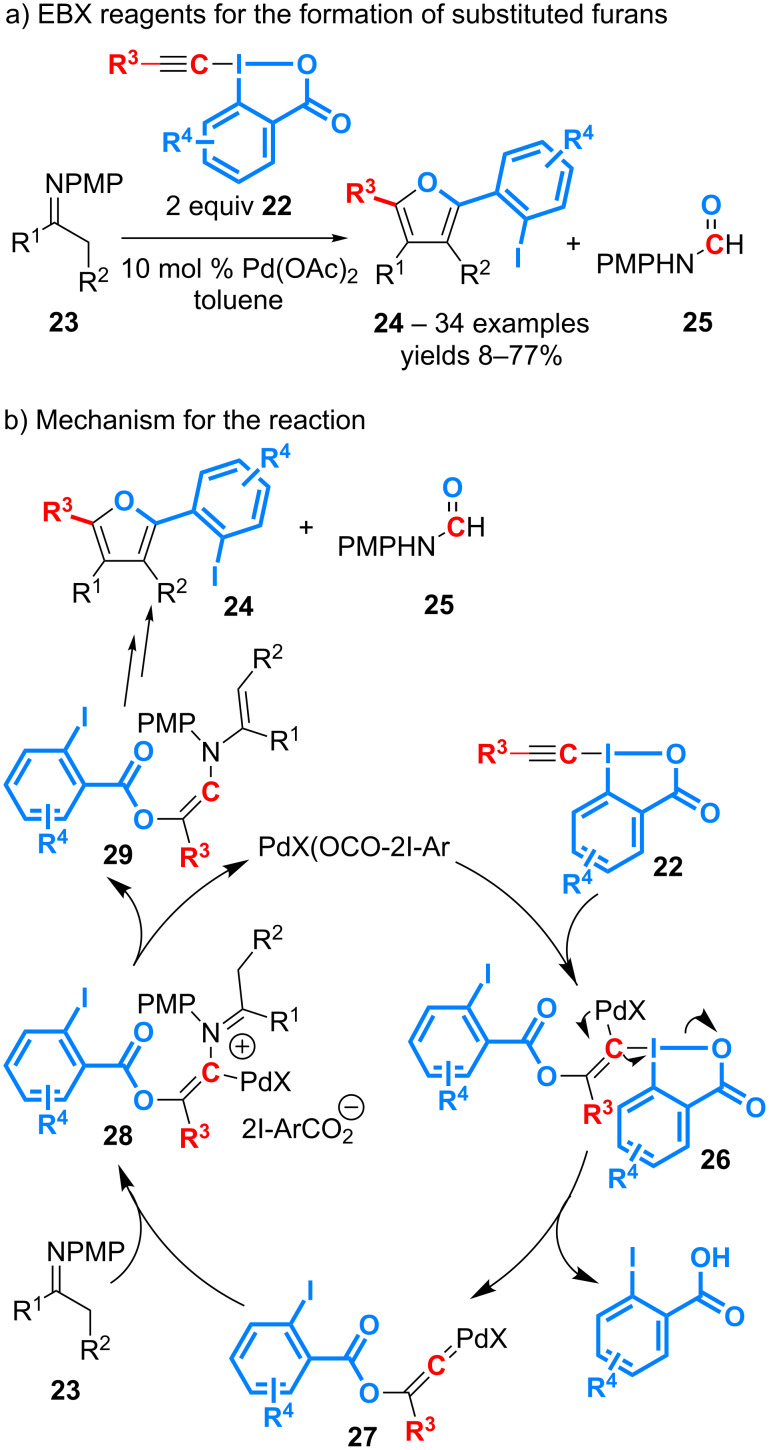
Alkynylation of *N*-(aryl)imines with EBX for the formation of furans.

Ethynylbenziodoxolone (EBX) is a powerful reagent to perform the electrophilic alkynylation of various functional groups such as carbonyl derivatives, thiols, arenes and heteroarenes [43–44]. A first solution to value the 2-iodobenzoic moiety released from EBX has arisen from the reaction of derivatives **22** with *N*-(aryl)imines **23** in the presence of 10 mol % of Pd(OAc)_2_, which gives access to tri- or tetrasubstituted furans **24** and *N-*(aryl)formamides **25** (Scheme 10a) [45]. The scope of the overall transformation is wide both in terms of imines and EBX reagents.

Several isotope-labelling experiments have allowed for proposing a mechanism for this complex transformation (Scheme 10b). The latter would first involve the addition of a Pd(II)-2-iodobenzoate species to the triple bond of **22** to give the intermediate **26**, followed by the reductive elimination of the trivalent iodine motif to afford the palladium-vinylidene **27**. This would undergo a nucleophilic addition of the imine and a subsequent proto-demetallation to give enamine **29**. A series of rearrangements including the cleavage of the triple bond and the fragmentation of the carboxylate unit, would finally lead to the furans **24** and the formamide **25**.

**Scheme 11:**
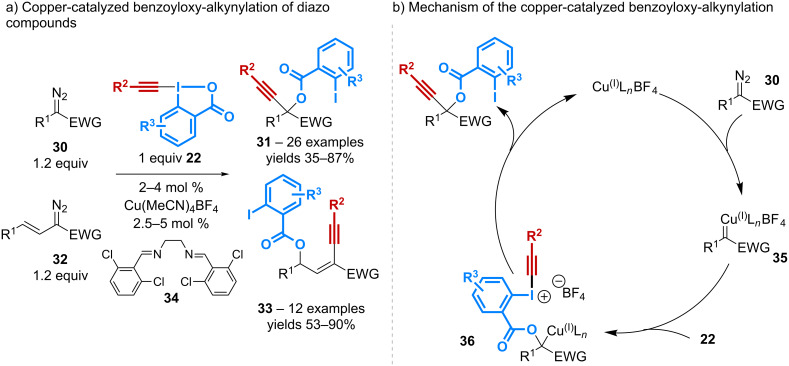
Catalytic benzoyloxy-alkynylation of diazo compounds.

The group of Waser has discovered an atom-economical multi-component process between alkynylbenziodoxolones **22** and diazo compounds, which is catalyzed by the copper(I) complex [Cu(MeCN)_4_]BF_4_. The reaction gives access to versatile building blocks while generating only N_2_ as side-product (Scheme 11a) [46]. Worth of mention is the use of a 1,2-diimine ligand **34** that is crucial to obtain good conversions.

Compounds **31** resulting from the *gem*-addition of the benzoate motif and the alkynyl group are obtained from various acceptor or donor–acceptor diazo compounds **30**, while the use of vinyldiazo derivatives **32** leads to enynes **33** arising from the vinylogous addition of the carboxylate. Significantly, the benzoyloxy-alkynylation reaction can be applied to the late-stage modification of complex products such as steroids. On the other hand, both the alkyne and the iodoarene moiety can be modified through post-transformation reactions thereby increasing the molecular diversity accessible with this process. The mechanism of the reaction (Scheme 11b) would first involve the formation of the copper-carbene species **35**, to which the carboxylate of alkynylbenziodoxolone could add to afford the intermediate **36**. Final alkynyl transfer would give rise to the products **31** and **33**, however, the nature of this alkynylation step remains to be elucidated.

**Scheme 12:**
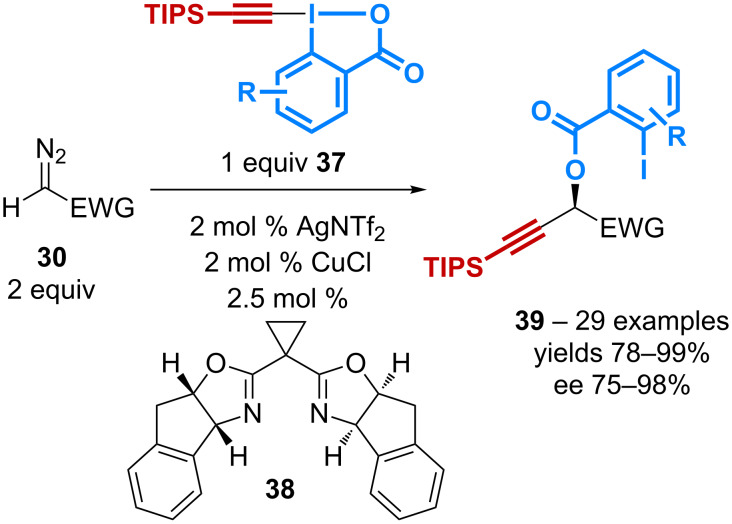
Catalytic asymmetric benzoyloxy-alkynylation of diazo compounds.

In a subsequent study, the same group has demonstrated the ability to perform the reaction with TIPS-EBX **37** in an enantioselective manner using the cyclopropylbisoxazoline ligand **38** (Scheme 12) [47]. Starting from various acceptor diazo compounds, the *gem*-addition of the carboxylate and the alkyne proceeds with ees of up to 98% to afford α-benzoyloxy propargylic derivatives **39**. Again the strategy can be applied to the late-stage modification of steroids with high levels of diastereoselectivity.

#### λ^3^-Iodane reagents: azidobenziodoxolone (ABX) and chlorobenziodoxolone

**Scheme 13:**
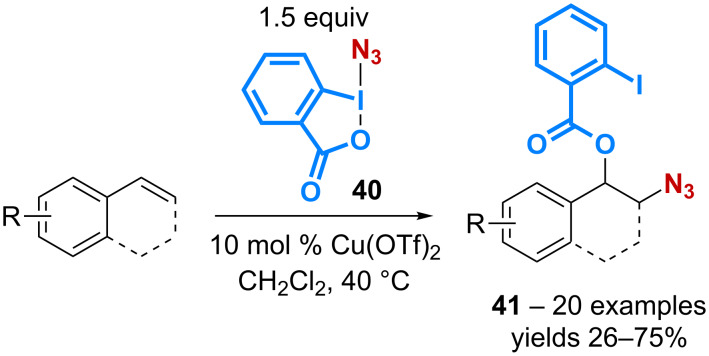
Catalytic 1,2-benzoyloxy-azidation of alkenes.

In a similar manner to the previously described catalytic benzoyloxy-trifluoromethylation using Togni’s reagent **5** (Scheme 4 and Scheme 5), the 1,2-benzoyloxy-azidation of alkenes can be performed in the presence of a copper catalyst with the azidobenziodoxolone ABX **40**. The reaction takes place in dichloromethane using the copper(II) complex Cu(OTf)_2_ as the catalyst thereby leading to the expected products **41** with yields in the 26–75% range (Scheme 13) [48]. Interestingly, an alkene diazidation reaction is observed by simply performing the transformation in DMSO and replacing Cu(OTf)_2_ by CuI as the catalyst.

**Scheme 14:**
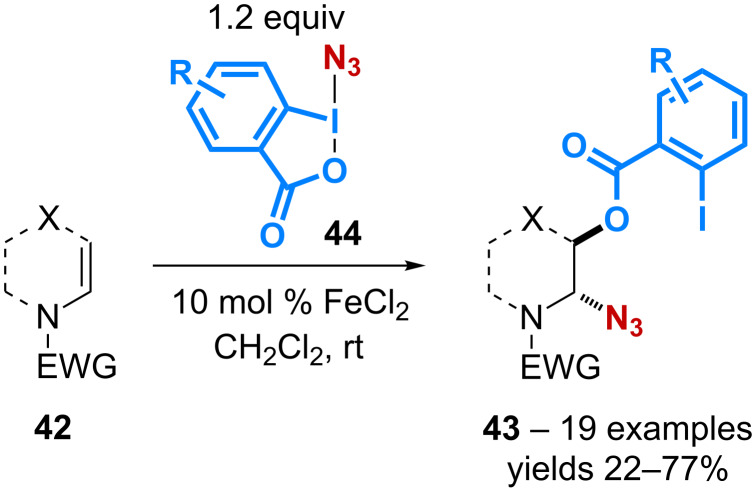
Catalytic 1,2-benzoyloxy-azidation of enamides.

Enamides **42** are also relevant substrates for the 1,2-benzoyloxy-azidation reaction. Based on a preliminary observation made during their study on catalytic trifluoromethylation of enamides [49], the group of Gillaizeau has reported the catalytic conversion of enamides with various ABX derivatives **44** [50]. A screening of various metal complexes has led the authors to demonstrate the superior ability of cheap FeCl_2_ to mediate the 1,2-addition thereby allowing the formation of the *trans*-products **43** with complete regio- and diastereocontrol (Scheme 14).

**Scheme 15:**
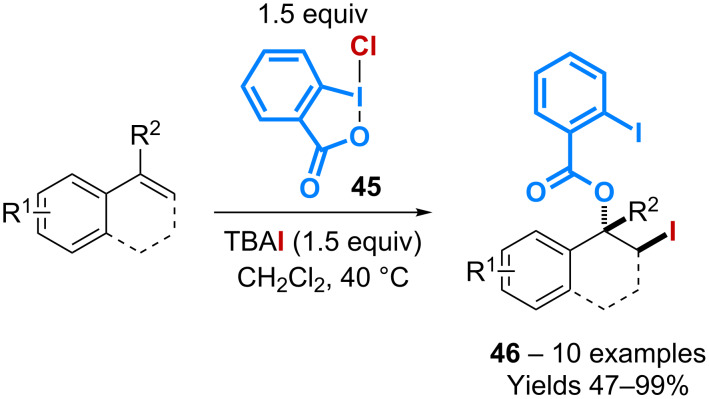
Catalytic 1,2-benzoyloxy-iodination of alkenes.

The group of Hamashima has reported that various 1,2-difunctionalizations of alkenes can occur with chlorobenziodoxolone **45** [51]. Oxychlorinated, dichlorinated, azidochlorinated and chlorothiocyanated products can thus be isolated mostly from styrenyl substrates. However, the treatment of **45** with tetra-butylammonium iodide followed by the addition of an alkene leads to the formation of products **46** resulting from a 1,2-benzoyloxyiodination and isolated with moderate to excellent yields (Scheme 15). The reaction is believed to take place through the formation of a hypoiodous species that activates the olefin via an iodonium intermediate.

### Tandem catalytic couplings

Diaryliodonium compounds, also named diaryl-λ^3^-iodanes, are stable, easy-to-handle crystalline solids, which have found numerous applications as arylating agents since their first use in the α-phenylation of 1,3-diones reported in the 60s [52–54]. Because of the hypernucleofuge nature of the Ar–I moiety in λ^3^-iodanes, their reactivity is higher than that of the corresponding aryl halides, a property that has been widely exploited to develop efficient transition-metal-catalyzed cross coupling reactions [55]. The latter, however, allow for transferring only one of the two aromatic motifs of the iodonium reagents. Several elegant catalytic domino reactions, accordingly, have been recently designed to incorporate both aryl groups into the products.

#### Cyclic diaryl-λ^3^-iodanes

**Scheme 16:**
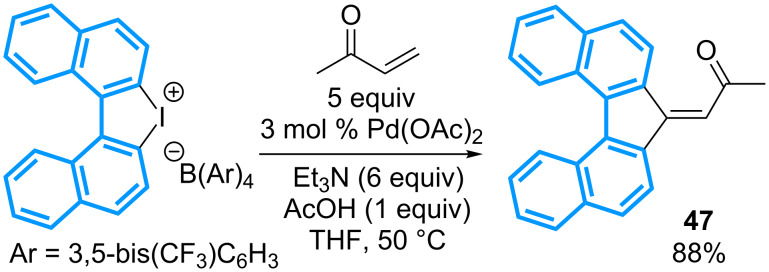
Seminal study with cyclic diaryl-λ^3^-iodane.

Cyclic diaryl-λ^3^-iodanes have been extensively studied for the preparation of complex polycyclic structures following the application of catalytic domino reactions. The group of Hayashi has reported in 2004 the first example of the transformation of a cyclic diaryl-λ^3^-iodane, for which both Ar–I bonds are used in a palladium cross-coupling reaction [56]. It relies on a double Heck reaction performed with methyl vinyl ketone in the presence of Pd(OAc)_2_ in THF, and affords the dibenzoalkylidenefluorene **47** in an excellent 88% yield (Scheme 16).

**Scheme 17:**
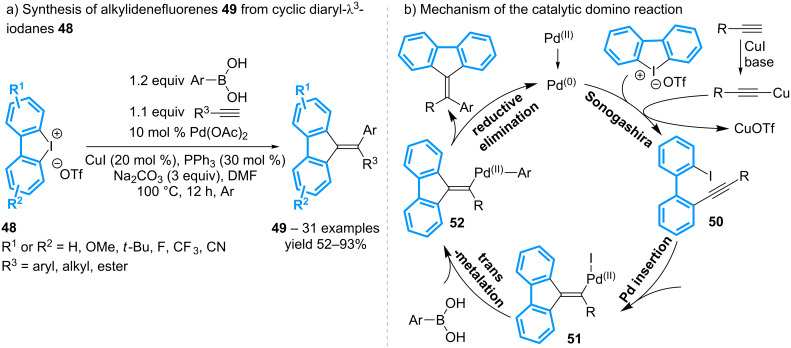
Synthesis of alkylidenefluorenes from cyclic diaryl-λ^3^-iodanes.

Ten years later, Huang, Wen and co-workers have demonstrated that, in the presence of both a terminal alkyne and a boronic acid, various cyclic diaryl-λ^3^-iodanes undergo a transition-metal catalyzed cascade reaction to afford alkylidenefluorenes **49** (Scheme 17) [57]. In terms of mechanism, a Pd(0)/Cu(I)-catalyzed Sonogashira coupling reaction from the iodonium salt **48** delivers a 2-alkynyl-2’-iodoarene **50** that, then, cyclizes to **51** via insertion of the Pd(0) species into the iodoarene moiety and migratory addition into the proximal alkyne. Transmetalation of the vinylpalladium with the boronic acid and reductive elimination finally leads to alkylidenefluorenes **49**. This multicomponent strategy allows the variation of the alkyne, the boronic acid and the diaryliodonium salts, but the use of non-symmetrical diaryl-λ^3^-iodanes raises the issue of regioselectivity.

**Scheme 18:**
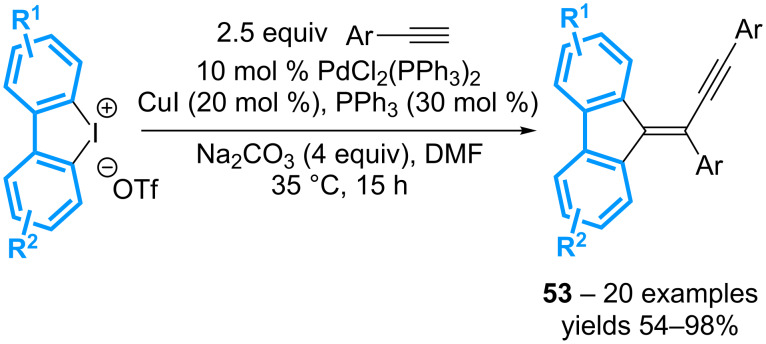
Synthesis of alkyne-substituted alkylidenefluorenes.

This strategy has been then extended to the preparation of alkyne-substituted alkylidenefluorenes **53** by replacing the arylboronic acid with a second equivalent of the terminal alkyne and performing the reaction at 35 °C (Scheme 18) [58].

Importantly, the reaction with non-symmetrical diaryl-λ^3^-iodanes gives the corresponding products with high regioselectivities, when an *o*-methyl substituent is present on one aromatic ring. The reaction can also be performed sequentially with the isolation of the iodoaryl intermediate, which can be resubmitted to the cyclization conditions in the presence of a different terminal alkyne, or an activated alkene.

**Scheme 19:**
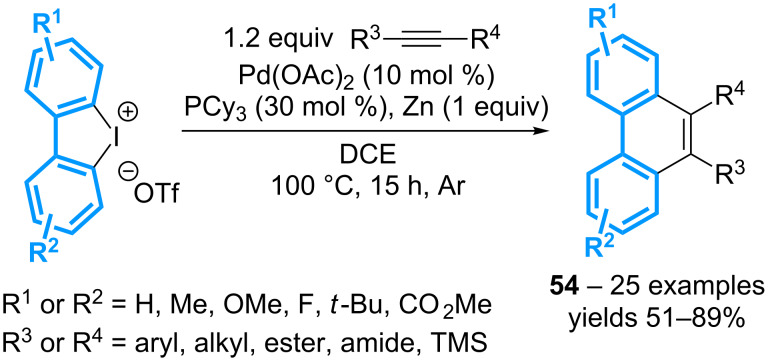
Synthesis of phenanthrenes from cyclic diaryl-λ^3^-iodanes.

The same authors have then showed that the reaction of cyclic diaryl-λ^3^-iodanes in the presence of internal alkynes and the catalytic system Pd(OAc)_2_-PCy_3_ affords functionalized phenanthrenes **54** in moderate to good yields (Scheme 19) [59].

**Scheme 20:**
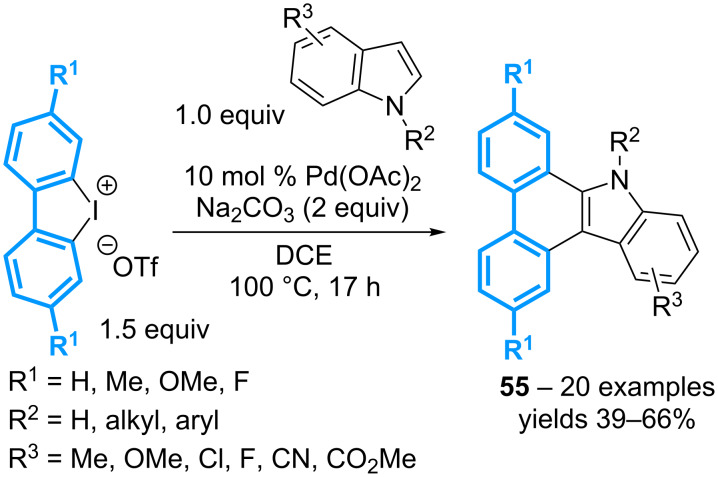
Synthesis of dibenzocarbazoles from cyclic diaryl-λ^3^-iodanes.

Indoles also are relevant substrates for the tandem arylation with cyclic diaryl-λ^3^-iodanes, allowing the preparation of dibenzocarbazoles **55** in moderate yields (Scheme 20) [60]. The reaction is catalyzed simply by Pd(OAc)_2_ in the absence of any ligands, and tolerates a variety of substituents on both the indole and the iodonium salt. However, the atom-economy of the reaction is limited by the need to use the iodonium salts in slight excess (1.5 equivalents).

**Scheme 21:**
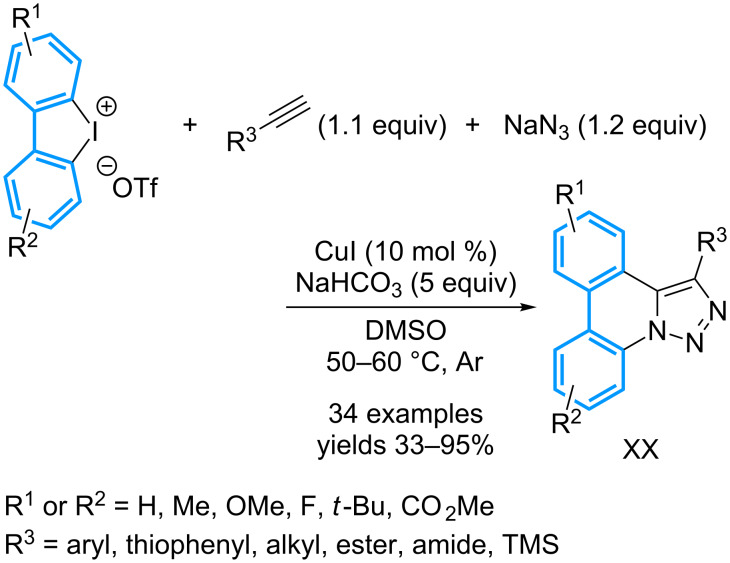
Synthesis of triazolophenantridines from cyclic diaryl-λ^3^-iodanes.

Another multicomponent reaction has been designed for the preparation of triazolophenanthridines **56** that are obtained in a one-pot manner by combination of a cyclic diaryl-λ^3^-iodane with sodium azide and a terminal alkyne, in the presence of 10 mol % of CuI (Scheme 21) [61]. The expected triazolophenanthridines were generally isolated in good to excellent yields, but the presence of strong electron-donating or withdrawing groups on the phenylacetylene moiety proved to be detrimental to the conversion. In addition, mixtures of regioisomeric products are generally obtained starting from non-symmetrical diaryl-λ^3^-iodanes. A series of reactions has been performed to gain insight into the mechanism that first involves the formation of a 2’-iodobiphenyl-2-azide promoted by the copper complex. The latter then catalyzes an intermolecular [3 + 2] cycloaddition with the alkyne. Finally, the copper-triazole moiety inserts intramolecularly into the second Ar–I bond, allowing a ring closure after reductive elimination.

**Scheme 22:**
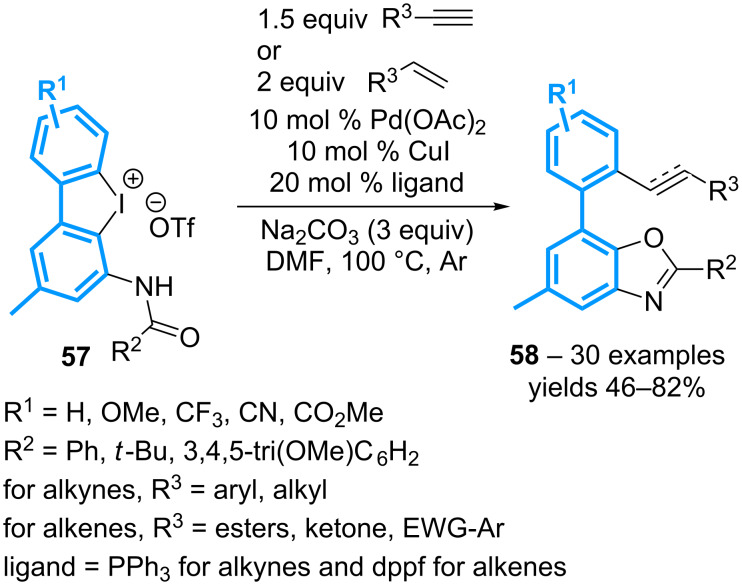
Synthesis of functionalized benzoxazoles from cyclic diaryl-λ^3^-iodanes.

Starting from the *ortho-N*-(acyl)diaryl-λ^3^-iodanes **57**, a combination of copper and palladium catalysis, in the presence of a phosphine ligand, induces the internal *O*-arylation of the proximal amide moiety, followed by a subsequent metal-catalyzed coupling-reaction with the resulting Ar–I motif (Scheme 22) [62]. Hence aryl- and alkyl-substituted terminal alkynes can be coupled via a Sonogashira reaction when PPh_3_ is used as ligand, while the use of diphenylphosphinoferrocenyl ligand (dppf) allows the Heck-type coupling of acrylates, vinyl ketones and electron-poor styrene derivatives. This relayed strategy can therefore be applied to non-symmetrical cyclic diaryliodonium species, thereby affording a library of functionalized benzoxazoles **58** with complete regiocontrol.

**Scheme 23:**
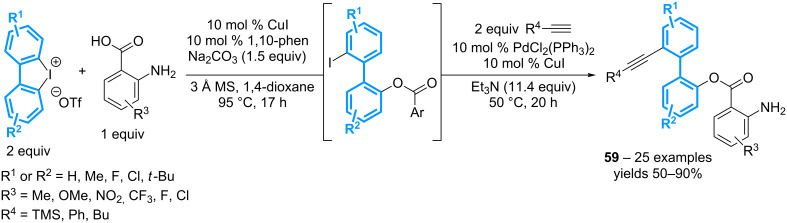
Sequential difunctionalization of cyclic diaryl-λ^3^-iodanes.

The group of Zhang has developed a one-pot procedure for the sequential difunctionalization of cyclic diaryl-λ^3^-iodanes (Scheme 23) [63]. The first step relies on the copper-catalyzed coupling between an anthranilic acid derivative and the biphenyl moiety. The resulting iodoarene product is then submitted to a Sonogashira coupling reaction, allowing the one-pot preparation of a library of biphenyl products **59** in moderate to good yields. Nevertheless, a two-fold excess of the iodonium triflate is needed to secure a good conversion, a point that limits again the atom-economy of the overall process.

**Scheme 24:**
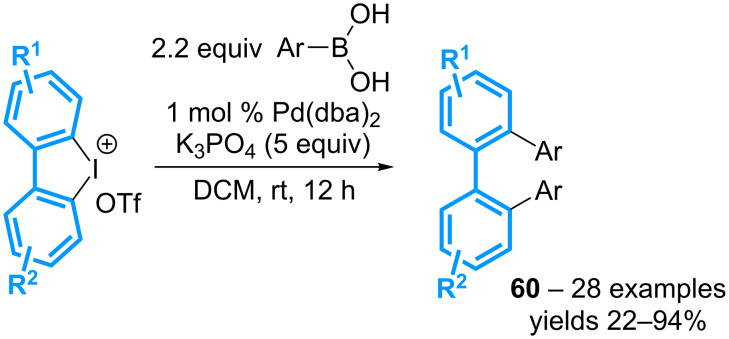
Double Suzuki–Miyaura coupling reaction of cyclic diaryl-λ^3^-iodanes.

Similarly, the group of Liu has described the double Suzuki–Miyaura coupling reaction of cyclic diaryl-λ^3^-iodanes with various aryl- and heteroarylboronic acids for the formation of *o*-tetraaryls **60** (Scheme 24) [64]. The reaction generally requires only 1 mol % of Pd(dba)_2_ to afford the expected products in good to high yields. It is worth mentioning that a comparative test starting from a 2,2’-diiodobiaryl has led to a much lower yield when compared to that obtained from the corresponding diaryl-λ^3^-iodane.

**Scheme 25:**
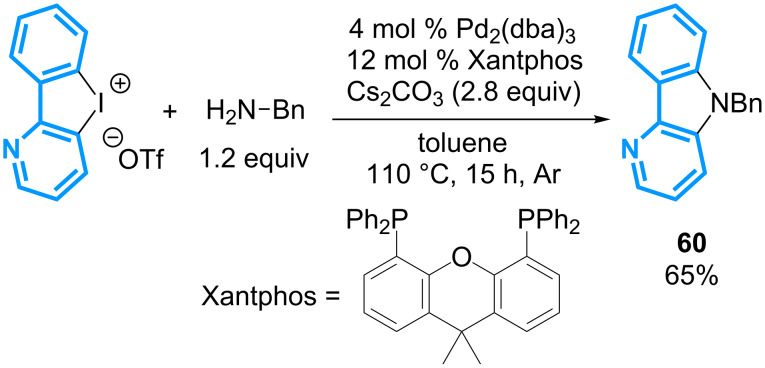
Synthesis of a δ-carboline from cyclic diaryl-λ^3^-iodane.

In 2011, the group of Detert has reported the first example of a palladium-catalyzed double C–N bond formation starting from the cyclic (phenyl)(pyrido)-λ^3^-iodane **61** (Scheme 25) [65]. The reaction requires the use of 4 mol % of Pd_2_(dba)_3_, 12 mol % of Xantphos, 2.8 equivalents of Cs_2_CO_3_ and 1.2 equivalents of benzylamine to afford the corresponding δ-carboline in a 65% yield.

**Scheme 26:**
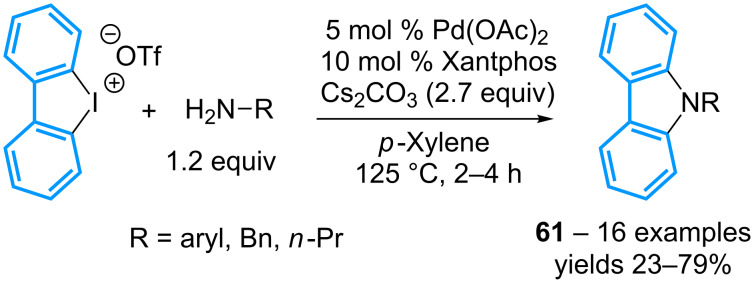
Synthesis of *N*-(aryl)carbazoles from cyclic diaryl-λ^3^-iodanes.

Two years later, the group of Nachtsheim has designed a similar strategy for the preparation of *N*-(aryl)carbazoles **61** from cyclic diaryl-λ^3^-iodanes (Scheme 26) [66]. The palladium phosphine ligand plays a crucial role as a bidentate ligand with a bite angle greater than 100° such as DPEphos (104°) or Xantphos (108°) significantly improves the yields. The reaction applies to a series of anilines and aliphatic amines, but electron-poor anilines afford better results than their electron-rich congeners, while a hindered amine (*t*-BuNH_2_) completely inhibits the reaction.

**Scheme 27:**
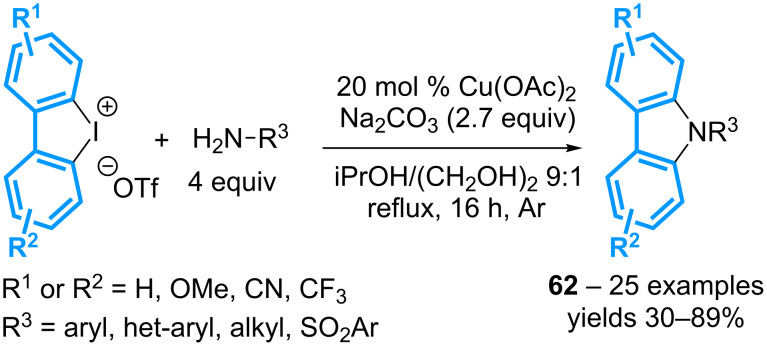
Synthesis of carbazoles from cyclic diaryl-λ^3^-iodanes.

Shortly after, a similar synthesis of carbazoles involving Cu(OAc)_2_ as the catalyst in the presence of ethylene glycol both as a ligand and the co-solvent has been reported (Scheme 27) [67]. A variety of amines such as anilines, sulfonamides and aliphatic amines has been utilized though in large excess. But in contrast to the previous method, electron-rich anilines proved to be better candidates for this reaction than the electron-poor analogs.

**Scheme 28:**
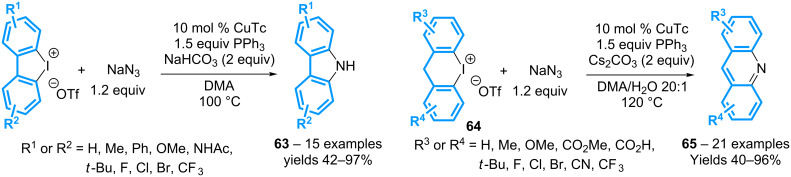
Synthesis of carbazoles and acridines from cyclic diaryl-λ^3^-iodanes.

The group of Jiang has very recently described a complementary method for the synthesis of carbazoles by combining cyclic diaryl-λ^3^-iodanes with sodium azide in the presence of copper(I) thiophene-2-carboxylate (CuTc) and triphenylphosphine. The reaction affords the expected of N–H free derivatives **63** in moderate to high yields, irrespective of the nature of the substituents (Scheme 28) [68]. The scope of the reaction has been extended to 6-membered ring cyclic diaryl-λ^3^-iodanes **64**. The acridines **65**, hence, have been obtained with similar yields.

**Scheme 29:**
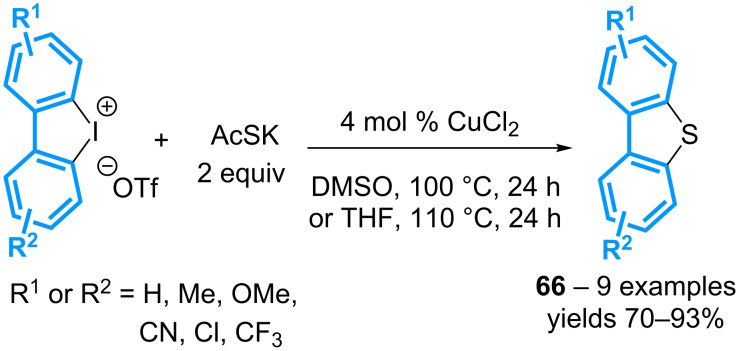
Synthesis of dibenzothiophenes from cyclic diaryl-λ^3^-iodanes.

The group of Shimizu has reported the formation of dibenzothiophenes **66** following the reaction of cyclic diaryl-λ^3^-iodanes with potassium thioacetate as the sulfur donor, and CuCl_2_ as the catalyst (Scheme 29) [69]. The reaction can be performed either in THF or DMSO, affording both symmetrical and non-symmetrical products in good to excellent yields.

**Scheme 30:**
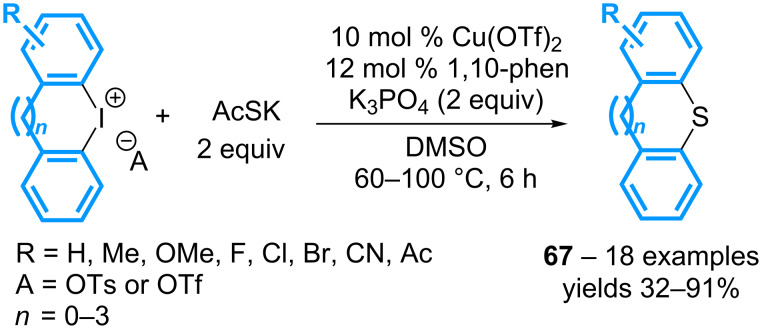
Synthesis of various sulfur heterocycles from cyclic diaryl-λ^3^-iodanes.

The scope of the reaction has been extended by using a combination of 10 mol % of Cu(OTf)_2_, 12 mol % of 1,10-phenanthroline, and 2 equivalents of potassium phosphate, as reported by the group of Jiang (Scheme 30) [70]. Six- to eight-membered sulfur heterocycles **67** can thus be isolated from cyclic diaryl-λ^3^-iodanes. More significantly, the reaction can be applied to acyclic diaryl-λ^3^-iodanes (vide infra).

**Scheme 31:**
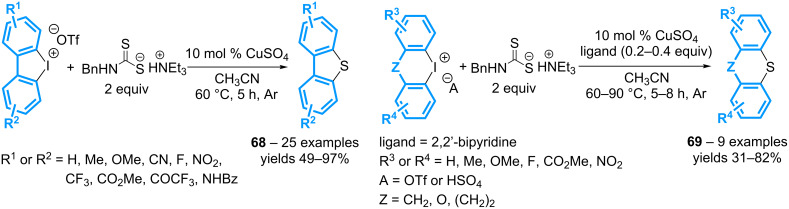
Synthesis of dibenzothioheterocycles from cyclic diaryl-λ^3^-iodanes.

A similar approach involving the *N*-benzyldithiocarbamate-triethylamine salt as the sulfur source and copper sulfate as the catalyst also provides dibenzothiophenes isolated generally in good to excellent yields, irrespective of the nature of the substituents (Scheme 31) [71]. The transformation has been extended to other cyclic diaryl-λ^3^-iodanes by using 2,2’-bipyridine as the copper ligand, allowing the preparation of the corresponding thioxanthenes, phenoxathiines and dibenzothiepines in moderate to good yields.

**Scheme 32:**
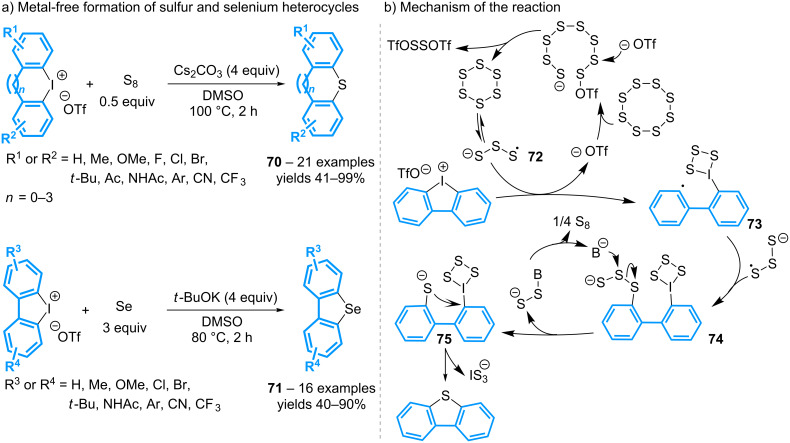
Synthesis of dibenzosulfides and dibenzoselenides from cyclic diaryl-λ^3^-iodanes.

A metal-free alternative to the previous methods of formation of sulfur heterocycles **70** has been reported by using elemental sulfur in the presence of cesium carbonate (Scheme 32) [72]. The strategy can be extended to the synthesis of the cyclic selenium analogs **71** by utilizing elemental selenium and potassium *tert*-butoxide. Several substituted 5- to 8-membered ring π-conjugated systems are isolated in moderate to high yields. Mechanistic investigations for the iodine/sulfur exchange have led to propose the initial formation of the trisulfur radical anion S_3_^•–^ **72**, which adds to the diaryliodonium moiety thereby inducing the formation of the aryl radical **73**. The latter then couples with another trisulfur radical anion to give the intermediate **74**. After the base-promoted formation of the corresponding thiophenol anion **75**, a cyclization delivers the expected product **70** by displacement of the iodine moiety.

**Scheme 33:**
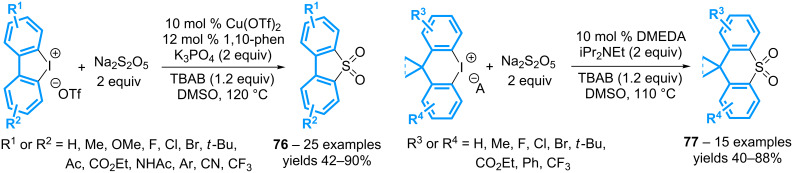
Synthesis of dibenzosulfones from cyclic diaryl-λ^3^-iodanes.

The same group has then developed a radical method to access the corresponding sulfones from cyclic diaryl-λ^3^-iodanes (Scheme 33) [73]. The reaction is catalyzed by a 1,10-phenanthroline-copper complex and affords the dibenzothiophene-5,5-dioxides **76** in moderate to high yields. The transformation has been further extended to the synthesis of a new series of molecules **77**, where the central thiophene moiety is replaced by a 6-membered heterocycle, which is substituted by a *gem*-dimethyl or a cyclopropyl group. In this case, the reaction can be performed under metal-free conditions in the presence of a catalytic amount of dimethylethylenediamine.

#### Linear diaryl-λ^3^-iodanes

**Scheme 34:**
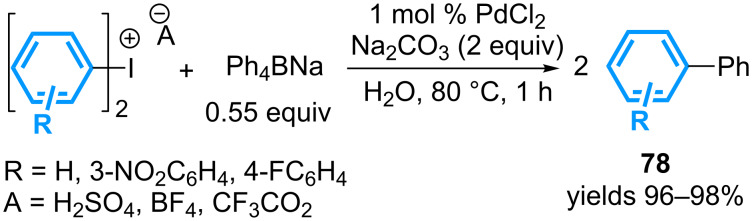
Seminal study with linear diaryl-λ^3^-iodanes.

When compared to cyclic diaryl-λ^3^-iodanes, the linear analogues inherently raise a more challenging issue to address in terms of sustainability, particularly in the case of non-symmetrical reagents. As mentioned in the introduction, the first study documenting the coupling of both aromatic groups of a diaryl-λ^3^-iodane has been reported in 1995 by Bumagin and co-workers [25]. One equivalent of symmetrical diaryl-λ^3^-iodanes could be engaged in palladium-catalyzed cross-coupling reactions with 0.55 equivalent of sodium tetraphenylborate to afford 2 equivalents of the corresponding biphenyl products in nearly quantitative yields (Scheme 34). It is assumed that the first cross coupling reaction with the iodonium salt liberates an aryl iodide moiety, available for the second palladium-catalyzed coupling reaction.

**Scheme 35:**
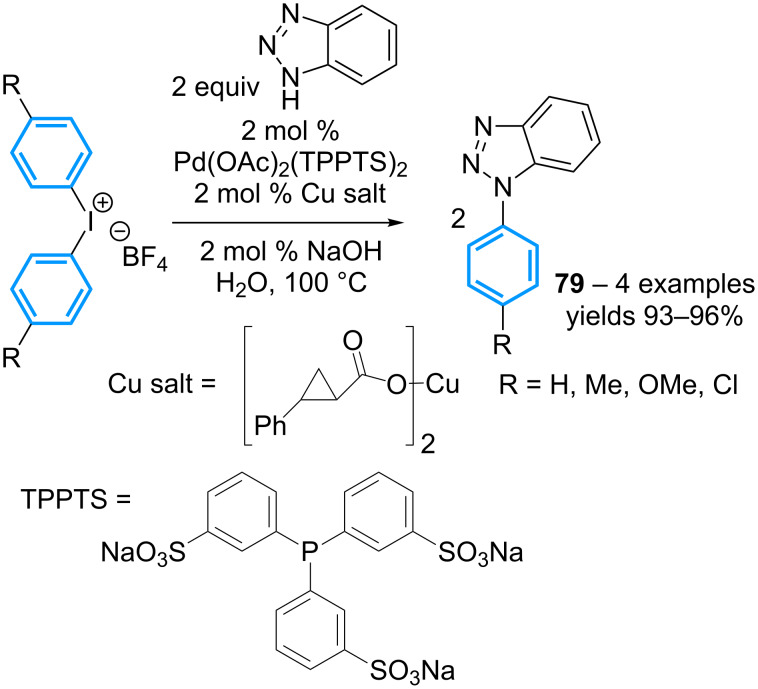
*N*-Arylation of benzotriazole with symmetrical diaryl-λ^3^-iodanes.

Three years later, the high yielding *N*-arylation of 1*H*-1,2,3-benzotriazole (BTA), utilizing symmetrical diaryl-λ^3^-iodanes as two-fold aryl donors has been reported in the presence of Pd(OAc)_2_ and TPPTS as a water-soluble ligand, and copper(II) phenylcyclopropylcarboxylate (Scheme 35) [74]. Noteworthy, it is mentioned that Ar–I fails to furnish the *N*-arylation products under the same reaction conditions, leading the authors to conclude that this suggested intermediate remains in the coordination sphere of the palladium catalyst.

**Scheme 36:**
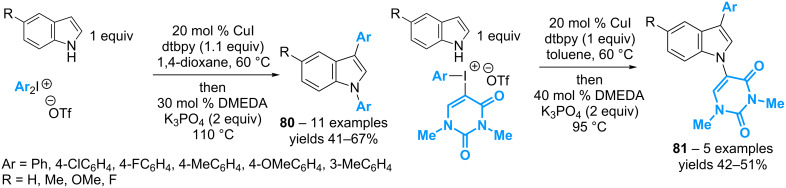
Tandem catalytic C–H/N–H arylation of indoles with diaryl-λ^3^-iodanes.

In 2015, the group of Greaney has described a tandem copper-catalyzed C_3_–H/N–H arylation of indoles with diaryl-λ^3^-iodanes (Scheme 36) [75]. The reaction first involves the use of 20 mol % of CuI and 1.1 equivalents of di-*tert*-butylpyridine (dtbpy) to convert the indole to the C_3_–H arylated product. The released Ar–I building block, then, can be engaged in the subsequent step of N–H arylation by adding 30 mol % of dimethylethylenediamine (DMEDA) and potassium phosphate to the same pot. Symmetrical diaryl-λ^3^-iodanes afford the diarylated indoles **80** with yields ranging from 41% to 67%. More significantly, whereas non-symmetrical diaryl-λ^3^-iodanes based on electron poor/rich aryl moieties do not provide good levels of regiocontrol in the tandem process, the use of (aryl)(dimethyluracyl)-λ^3^-iodanes allows for obtaining difunctionalized indoles of type **81** resulting from the regioselective introduction of the dimethyluracyl group at the N–H position.

**Scheme 37:**
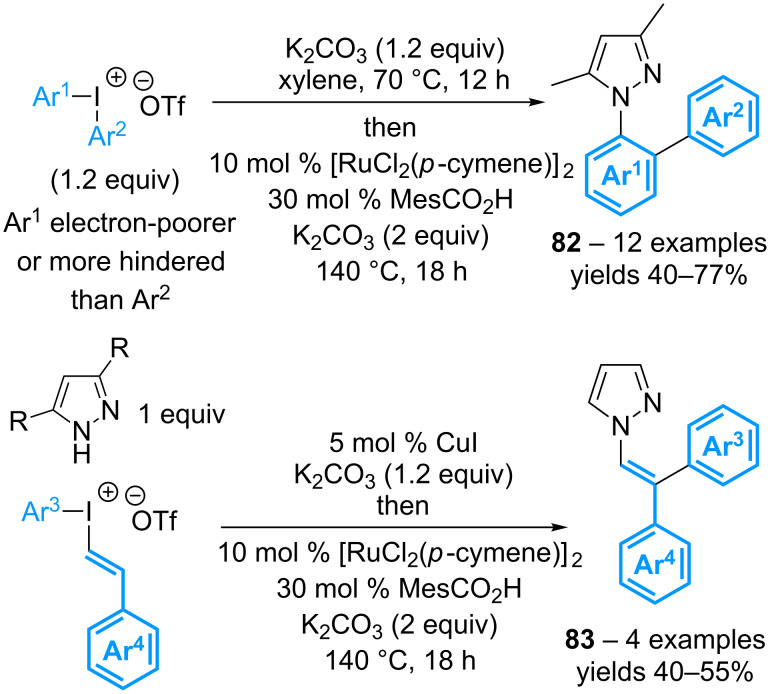
Tandem *N*-arylation/C(sp^2^)–H arylation with diaryl-λ^3^-iodanes.

The same group has then reported a remarkable domino process for the introduction of both aromatic rings of diaryl-λ^3^-iodanes, though a small excess of this reagent is needed to obtain good yields. An initial *N*-arylation reaction of 3,5-dimethylpyrazole is performed in the presence of potassium carbonate, in xylene at 70 °C, releasing 1 equivalent of aryl iodide. The latter is then used for a sequential ruthenium-catalyzed *ortho*-C–H functionalization directed by the pyrazole group (Scheme 37) [76]. Starting from non-symmetrical diaryl-λ^3^-iodanes, the electron-poorest or more sterically hindered aromatic group is first transferred to the 3,5-dimethylpyrazole. The reaction has been extended to 1,2,3-triazoles, benzotriazole, and pyrazole. In the latter case, the use of (styryl)(aryl)-λ^3^-iodanes has also proved to be possible, with the styryl moiety being selectively transferred in the first step. The following step of C–H activation then gives access to 2,2-diarylated enamines **83** isolated as a single isomer with respect to the alkene geometry.

**Scheme 38:**
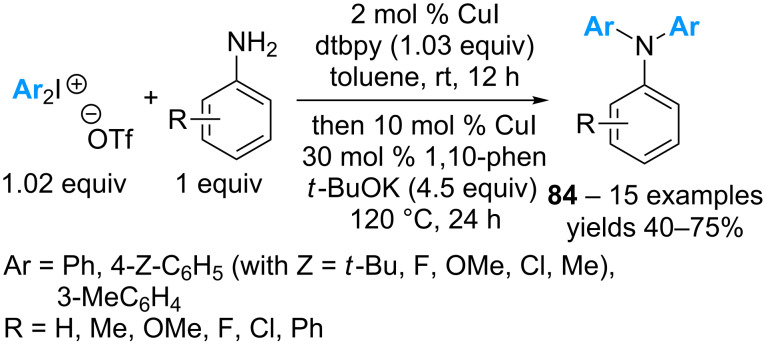
Catalytic intermolecular diarylation of anilines with diaryl-λ^3^-iodanes.

While the diarylation of anilines with cyclic diaryl-λ^3^-iodanes for the formation of *N*-arylated carbazoles has been described in 2013 [66–67], the truly intermolecular diarylation of anilines with linear reagents has been described only in 2017 by the group Greaney [77]. Except for anilines bearing strong electron-withdrawing substituents (*p*-NO_2_), the triarylated amines **84** can be obtained in moderate to good yields, irrespective of the substitution of the aniline and the diaryl-λ^3^-iodane (Scheme 38). However, the scope of the reaction is limited to symmetrical diaryl-λ^3^-iodanes as non-symmetrical reagents afford mixtures of *N*-arylated products in the first step of the tandem process.

**Scheme 39:**
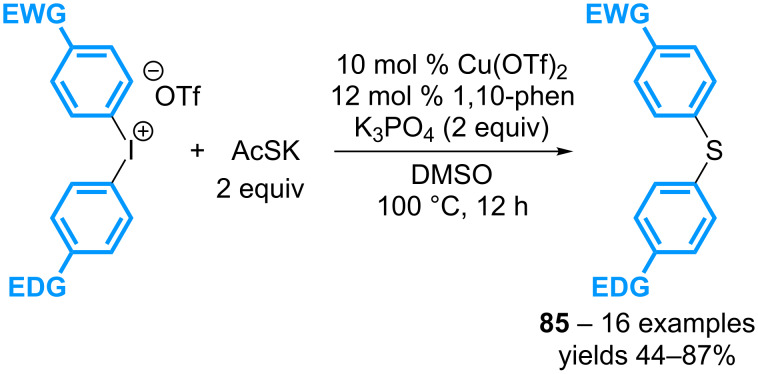
Catalytic synthesis of diarylsulfides with diaryl-λ^3^-iodanes.

As mentioned in the Scheme 30, the group of Jiang has reported the formation of cyclic diarylsulfides from the corresponding cyclic diaryl-λ^3^-iodanes, utilizing potassium thioacetate as the sulfur source, in the presence of 10 mol % of Cu(OTf)_2_, and 12 mol % of 1,10-phenanthroline [70]. This transformation has been successfully applied to linear diaryl-λ^3^-iodanes (Scheme 39). Interestingly, symmetrical and non-symmetrical reagents bearing two electronically different aryl groups can be transferred to provide the corresponding diarylsulfide products **85**, a result that stands in contrast to the observation made by Greaney in his study with anilines described in Scheme 38.

#### Alternative to diaryl-λ^3^-iodanes for the α-arylation of carbonyl compounds

**Scheme 40:**
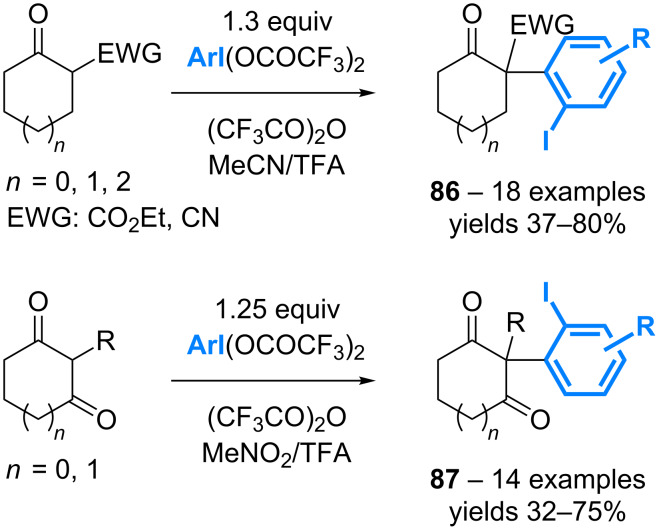
α-Arylation of enolates using [bis(trifluoroacetoxy)iodo]arenes.

In addition to their role as aryl donors in numerous metal-catalyzed couplings, diaryl-λ^3^-iodanes are also relevant reagents to perform the α-arylation of enolates. However, the reaction is again limited to the transfer of a single aryl group. As an alternative to address this issue of atom-economy, the group of Shafir has reported the metal- and base-free arylation of keto-esters and cyanoketones using [bis(trifluoroacetoxy)iodo]arenes ArI(OCOCF_3_)_2_ (Scheme 40) [78]. The reaction can also be applied to cyclic 1,3-diones with equal efficiency [79].

**Scheme 41:**
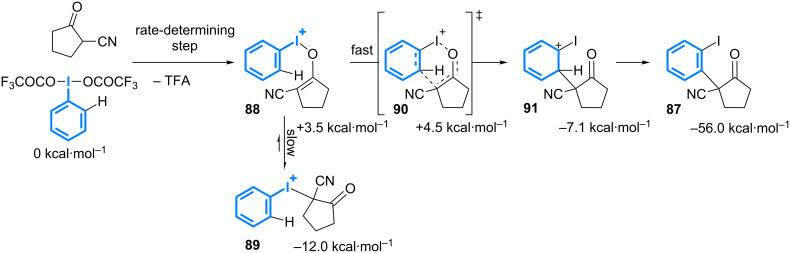
Mechanism of the α-arylation using [bis(trifluoroacetoxy)iodo]arene.

Mechanistic studies supported by DFT calculation have led to propose that the rate-determining step of the process would be the ligand exchange between TFA and the *O*-enolate (Scheme 41) [79]. The resulting cationic intermediate **88** could rapidly evolve through a [3,3] rearrangement. Even though the *C*-enolate **89** is more stable than its *O*-tautomer **88**, the rearrangement step seems to be much faster. The use of the radical scavenger TEMPO had no effect on the reaction outcome suggesting that the initial hypothesis is correct. By screening additives, it was shown that the hypervalent iodine could be quickly generated in situ by using Oxone as a terminal oxidant, thereby allowing for extending the scope of the reaction in terms of iodoarenes.

#### Tandem oxidation–catalytic couplings

**Scheme 42:**
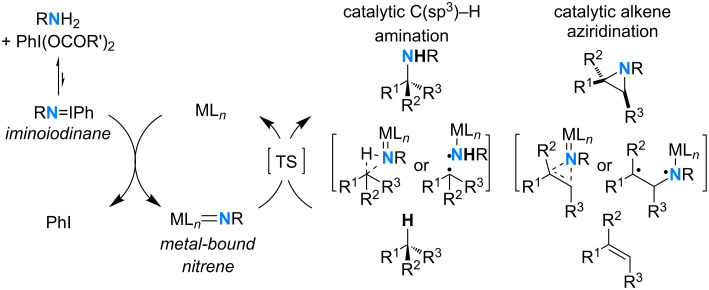
Catalytic nitrene additions mediated by [bis(acyloxy)iodo]arenes.

A large range of oxidation reactions can be performed with [bis(acyloxy)iodo]arenes best represented by the commercially available reagents PhI(OAc)_2_ and PhI(OCO*t-*Bu)_2_. These λ^3^-iodanes have been widely used in atom-transfer reactions, particularly for the generation of metal-bound nitrenes that are highly active species for the aziridination of alkenes and the direct amination of benzylic, allylic or tertiary C(sp^3^)–H bonds [80–85]. Seminal catalytic nitrene transfers mediated by λ^3^-iodanes [86–89] were described from iminoiodinanes of general formula PhI=NR that can be prepared mainly from sulfonamides [90]. However, the scope of catalytic C(sp^3^)–H amination and alkene aziridination reactions has been greatly enhanced following the discovery of practical procedures for the in situ generation of iminoiodinanes, a synthesis which is known to be troublesome [91–93]. According to these protocols and following the design of dirhodium(II) complexes, highly efficient catalytic nitrene transfers have been reported from carbamates [92], sulfamates [94–97], ureas and guanidines [98], sulfamides [99], hydroxylamine-derived sulfamates [100], carbamimidates [101], and sulfonimidamides [102–107]. These reactions involve the formation of a metal-bound nitrene that can insert into a C(sp^3^)–H bond or a π-bond via the asynchronous concerted addition of a singlet species or a stepwise radical pathway (Scheme 42) [83–84].

**Scheme 43:**
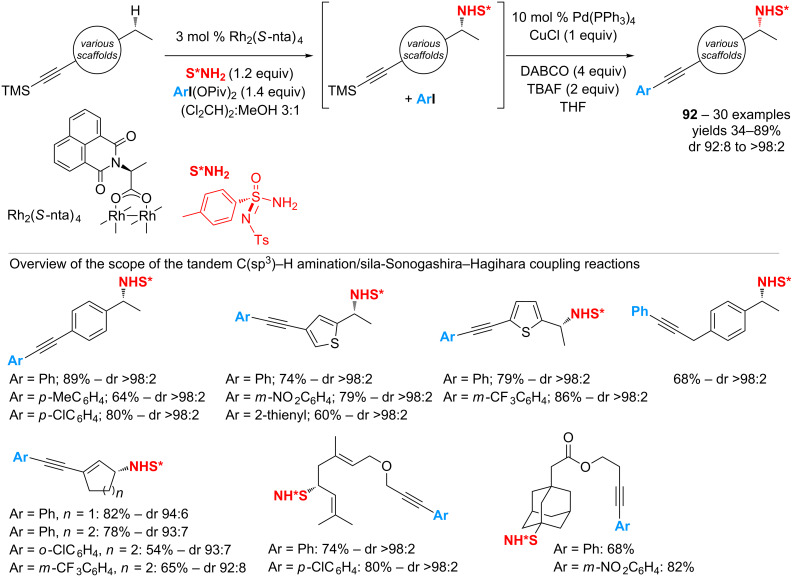
Tandem of C(sp^3^)–H amination/sila-Sonogashira–Hagihara coupling.

While these transformations cannot be performed under conditions catalytic in iodine, recent investigations have revealed the possibility to value the iodoarene side-product in a subsequent one-pot palladium-catalyzed cross-coupling reaction. The use of a sulfonimidamide (S*NH_2_), as a chiral nitrene precursor, in combination with the chiral dirhodium(II) complex Rh_2_(*S*-nta)_4_ has led to the discovery of intermolecular C(sp^3^)–H amination reactions that proceed from hydrocarbons used as the limiting components, with nearly quantitative yields and complete diastereoselectivity [102–105]. Such a highly efficient atom-transfer process has provided the opportunity to design a tandem of C–N and C–C bond-forming reactions that relies on an initial step of catalytic nitrene addition. The latter would release the iodoarene that could be coupled to a suitable functionality previously introduced on the starting material. The strategy has been first validated from TMS-protected alkyne derivatives via a tandem of C(sp^3^)–H amination/sila-Sonogashira–Hagihara coupling (Scheme 43) [108].

The overall process affords complex nitrogen-containing compounds **92** with very good yields and complete stereocontrol starting from benzylic, allylic and adamantyl substrates. In addition, the preparation of substituted [bis(acyloxy)iodo]arenes following the reaction of iodoarenes with sodium perborate enables the introduction of various aryl groups on the alkyne moiety.

**Scheme 44:**
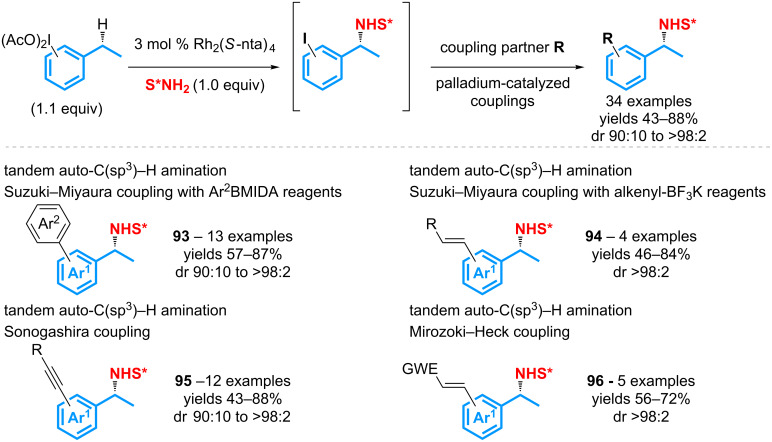
Tandem reaction using a λ^3^-iodane as an oxidant, a substrate and a coupling partner.

However, as a limitation of this study, the reaction allows for recycling at best 1 equivalent of the iodoarene while it involves the use of 1.4 equivalents of the λ^3^-iodane. Moreover, it requires the design of specific alkynyl substrates that reduces its scope. With the aim to address these issues, it has been demonstrated that, in addition to its role of oxidant and coupling partner, the λ^3^-iodane can simultaneously be used as the substrate for the C(sp^3^)–H amination reaction (Scheme 44) [109]. The catalytic auto-amination, thus, gives access to various iodoaminated intermediates that can be subsequently engaged in palladium-catalyzed Suzuki–Miyaura, Sonogashira, or Mizoroki–Heck cross-coupling reactions. Various aryl, alkenyl, or alkynyl substituents can thus be introduced in good to very good yields.

When compared to the previous strategy, not only this process enables to increase the molecular diversity, but it also displays a higher atom-efficiency as only 1.1 equivalents of the λ^3^-iodane are required to achieve good to excellent conversions. In addition, it should be pointed out that the tandem reactions based on the auto-amination process enables to address the issue of chemoselectivity in some cases. For example, the Suzuki–Miyaura coupling allows for introducing a pyridinyl or a furyl ring that are not compatible with the rhodium-catalyzed oxidizing amination reactions.

**Scheme 45:**
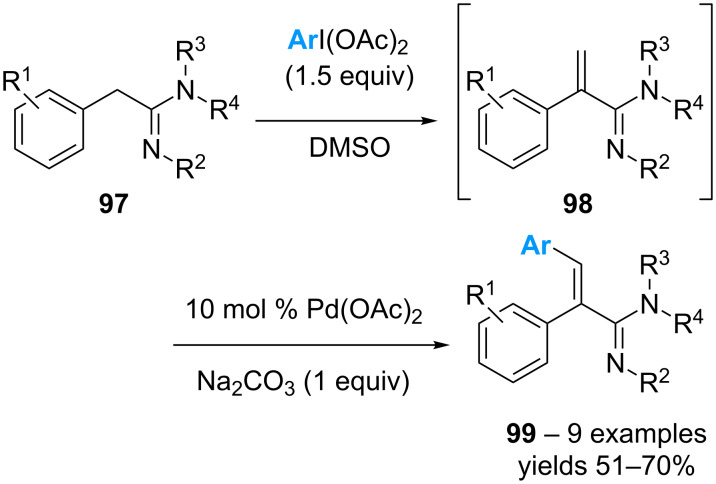
Synthesis of 1,2-diarylated acrylamidines with ArI(OAc)_2_.

In a similar manner, the group of Namitharan has very recently demonstrated that a one-pot palladium-catalyzed Heck coupling allows for transferring the aryl group of (diacetoxyiodo)arenes released after a metal-free methylenation reaction (Scheme 45) [110]. The latter that is performed by reacting PhI(OAc)_2_ with DMSO, applies to amidines **97** to afford the methylene intermediates **98** The following coupling leads to 1,2-diarylated acrylamidines **99** in good yields, but only starting from iodoarenes substituted by electron-donating groups.

## Conclusion

With the aim to reduce the formation of iodo-containing side-products, the design of tandem reactions that enable the incorporation of the aryl groups of λ^3^- or λ^5^-iodanes into the products, has emerged as a relevant versatile alternative to the solid-supported reagents and the iodine-catalyzed transformations. Most of the achievements reported in this context have been made through the application of modern transition-metal-catalyzed methods. Simple hypervalent iodine reagents can now be considered as valuable building blocks in the synthesis of both polyfunctionalized compounds and complex polycyclic skeletons. We believe that the application of this strategy could be a source of inspiration for the conception of new multicatalytic cascades that receive increasing attention in organic synthesis [111–112]. Extending their scope in terms of molecular diversity and complexity is expected from their application starting from other classes of λ^3^- or λ^5^-iodanes.
